# Evaluation of Outreach of Community Pharmacists in Public Health Services in Al-Jouf Region of Saudi Arabia: Findings and Implications

**DOI:** 10.3390/healthcare11162299

**Published:** 2023-08-15

**Authors:** Abdulaziz Ibrahim Alzarea, Yusra Habib Khan, Abdullah Salah Alanazi, Nasser Hadal Alotaibi, Sami I. Alzarea, Ziyad Saeed Almalki, Saad S. Alqahtani, Tauqeer Hussain Mallhi

**Affiliations:** 1 Department of Clinical Pharmacy, College of Pharmacy, Jouf University, Sakaka 72388, Saudi Arabia; yhkhan@ju.edu.sa (Y.H.K.); asdalananzi@ju.edu.sa (A.S.A.); nhalotaibi@ju.edu.sa (N.H.A.); 2 Health Sciences Research Unit, Jouf University, Sakaka 72388, Saudi Arabia; 3 Department of Pharmacology, College of Pharmacy, Jouf University, Sakaka 72388, Saudi Arabia; samisz@ju.edu.sa; 4 Department of Clinical Pharmacy, College of Pharmacy, Prince Sattam Bin Abdulaziz University, Al-Kharj 11942, Saudi Arabia; z.almalki@psau.edu.sa; 5 Department of Clinical Pharmacy, College of Pharmacy, King Khalid University, Abha 62529, Saudi Arabia; ssalqahtani@jazanu.edu.sa

**Keywords:** community pharmacist, public health services, Al-Jouf, community pharmacies, health promotion, Saudi Arabia

## Abstract

Background: Diversifying the conventional role of community pharmacists from dispensing to involvement in public health services could help in optimized patient care and ultimately good health practices. The current study aimed to ascertain the involvement of community pharmacists, barriers to involvement, their preparedness towards the provision of public health services in the future, and effective strategies to improve their existing role, especially in remote areas of the Kingdom of Saudi Arabia. Methods: A cross-sectional study was conducted in the Al-Jouf region of Saudi Arabia (KSA), between January to April 2023. A convenient sampling technique was used to recruit community pharmacists (CPs). A self-designed and validated questionnaire was used for data collection. The relative importance index (RII) was utilized to rank the barriers to participation in public health services. Data were subjected to statistical analysis using SPSS. Results: This study recruited 119 participants (mean age: 32.2 ± 7.9; male gender: 67.2%). Of these, 91.6% were involved in the provision of public health services at community pharmacies. Majority of CPs (n = 114/119, 95.8%) provided drug use-related written information to the patients, and the least practiced service was screening of dyslipidemia (n = 81; 68.1%). According to RII, the major barrier was the lack of time given by patients (RII: 0.812). Overall, the majority of the pharmacists (n = 94/119; 79%) were willing to provide public health services. Most of the CPs reported that empowerment through education and awareness (n = 100/119; 84%) is most effective strategy to enhance the involvement of pharmacists in public health services. Conclusions: Findings of the present study underscored the adequate participation of community pharmacists in public health activities. Further studies are required in other remote regions of KSA to get a clear insight into the overall participation of community pharmacists in public health services and generalize the findings.

## 1. Introduction

Apart from the conventional role of dispensing and compounding, pharmacist’s role has evolved as a vital part of the multi-disciplinary workforce providing extended services at the community level [1,2]. However, this paradigm shift in pharmacist’s role from dispensing to integrated involvement in public health services is pertinent, but limited to developed countries and needs further uplifting in other regions of the world [3]. These services at the community level include the provision of drug-related information, counseling regarding drug use, lifestyle modification, screening of chronic and infectious diseases, medication therapy management (MTM), drug interactions, potential and actual adverse drug reactions (ADRs), and awareness regarding immunization [4,5]. However, Community Pharmacists (CPs) are acknowledged as the most accessible healthcare professionals in the Kingdom of Saudi Arabia (KSA) [6,7,8,9].

Despite recommendations from World Health Organization (WHO) on pharmacists’ role in providing patient-oriented services, the participation of CPs in public health activities needs to be further strengthened by the relevant authorities around the globe [2]. If these recommendations are implemented, health benefits will be optimized, and patients will also benefit from CPs’ knowledge and skills [10]. The new pharmacy model Vision 2030 proposed by the Saudi government, envisaged the provision of ambulatory pharmaceutical care services through community pharmacies by expanding the role of CPs [11]. Further studies in different regions of KSA can ascertain the current practices and help with the road map to achieve this new model vision.

The Vision Saudi Arabia 2030 program, envisioning an effective and more integrated healthcare system, was launched by the government of KSA in 2016. Among the fundamental objectives of the programs to promote improved health services include facilitating access, quality, and efficiency of healthcare services, promoting health risk prevention, and reinforcing traffic safety [12]. Different strategical plans were devised by the Ministry of Health (MOH) to keep the program afloat and achieve its ultimate goal [13]. Keeping up with the strategies, a new pharmacy model for Vision 2030 in Saudi Arabia was also devised that was in line with the one, five, seven, and nine number strategies of the MOH plan [11]. The Saudi Vision 2030 program emphasizes public health services, and this program needs to be implemented in all of the KSA. To ensure the current status of this program, there is a need to assess the public health services provided in remote areas and barriers to ensure full coverage, other than only being implemented in big studies.

There is a scarcity of information regarding the involvement of CPs in public health services, all over KSA, and it is pertinent to ascertain their factual involvement, especially in remote regions like Al-Jouf. Present study aimed to bridge the literature gap and also answer the aforementioned research questions. Al-Jouf region was selected because it has limited access to healthcare facilities and healthcare professionals and is located in a remote region. The current study will provide an actual picture of the services in remote areas rather than taking into account the outcomes of studies in metropolitan cities reporting varying levels of CPs’ participation and hence requiring further investigation [10]. The rationale of the study was to bridge the literature gap regarding the problem statement. Few studies have been conducted regarding the role of pharmacists in community services in Saudi Arabia. Although good knowledge and preparedness of CPs in Saudi are previously reported, however, these studies were conducted mainly in cosmopolitan cities such as Jeddah, Makkah, Riyadh, and Madinah [14].

The present study aimed to ascertain the practices conducted in Al-Jouf, which is a remote region and comparatively has limited healthcare facilities, less developed infrastructure, and a smaller number of pharmacists as compared to other large cities of the countries. Due to these circumstances, very few pharmacists are attracted to this area, which will definitely impact the quantity and quality of the services provided by community pharmacists in this region. Moreover, authors belong to the Al-Jouf region. This study will further provide an outlook on the services in which CPs are involved in such remote regions compared to studies carried out in larger cities with sufficient resources and access to health facilities. This study also intended to identify the barriers to the participation, preparedness, and willingness of CPs towards the provision of additional services in the future, and recommendation of effective strategies to further improve the involvement of CPs in public health activities in Al-Jouf. To the best of our knowledge, previously conducted studies in smaller areas did not report the preparedness of the CPs to participate in such activities at the public health level [10]. The findings of the current study will fill the gap in the current literature, will reflect the factual involvement of CPs in Al-Jouf, and will pertinently provide effective strategies to overcome the barriers and enhance the participation of pharmacists in public health activities.

## 2. Materials and Methods

### 2.1. Ethical Approval

Prior to the commencement of the survey, the study was approved by the Local Committee on Bioethics (LCBE) of Jouf University (Reference no.: 7-10-43). Description of the study and its purpose was provided at the beginning of the survey. Informed consent was obtained from all the participants and their anonymity was maintained.

### 2.2. Study Design and Settings

**Figure 1:**
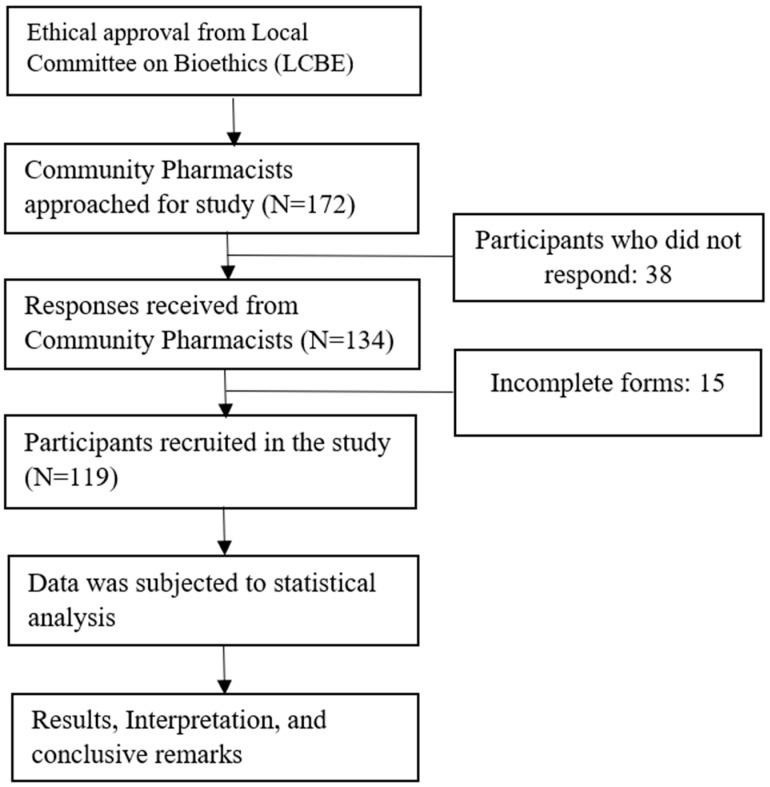
Study flow diagram.

A cross-sectional study was conducted among community pharmacists working in Al-Jouf province, Saudi Arabia. Data were collected over a period of four months (January to April 2022). Al-Jouf is one of the 13 provinces of KSA, having an estimated population of 0.5 million [15]. It is a less-developed province in the northern region sharing its border with Jordan, in comparison to the metropolitan provinces. Being a remote region, there is limited access to healthcare facilities as well as healthcare professionals including pharmacists. Inclusion criteria for this current study include: (1) a qualified pharmacist working in a community pharmacy, (2) willing to participate in the survey, (3) either male or female gender, (4) employee or self-employed, and (5) able to write and understand Arabic and English. Those participants who did not fulfill the criteria were excluded from the study. The flow diagram of the present study is shown in Figure 1.

### 2.3. Sampling Technique

A convenient sampling technique was used to collect the data from the three major cities of the Al-Jouf region, i.e., Sakaka, Dumat al-Jandal, and Qurayat. For data collection purposes, all the accessible pharmacies were identified and lists were prepared, regardless of the type of pharmacy (chain or single pharmacy). Investigators visited these community pharmacies in-person and collected data from the on-duty pharmacists. Data was collected on a paper-based study tool from those who consented to participate. Pharmacists who consented and were on duty were recruited in the survey by the investigators, ensuring to maintain anonymity.

### 2.4. Study Instrument

The data collection tool was self-designed with a literature review and consisted of close-ended questions [10,16,17,18]. The questionnaire was in English and Arabic, keeping in view the multi-cultural participants of KSA working at the community level and their better command of the language to avoid any inaccuracy. The questionnaire was reviewed by a panel of experts from different health professions for its content validity. After the content validity, recommended changes were made in the questionnaire before piloting. For face validity, and to assess comprehensibility, the study tool was pre-tested in a small sample (n = 30) for further clarity. Feedback from the CPs in the pilot study, not included in the final survey, was instilled into the final data collection tool. A Cronbach alpha value of 0.69 indicated the adequate reliability and consistency of the questionnaire.

The questionnaire consisted of five sections evaluating different aspects of CPs’ involvement in public health services. Section I consisted of 10 questions related to the demographics of the study participants. Section II comprised of 17 close-ended questions assessing the involvement of CPs in different services in public health, classified under five different categories. Section III contained 14 questions assessing barriers to pharmacists’ involvement in public health services on a 5-point Likert scale (1 = strongly agree, 2 = agree, 3 = neutral, 4 = disagree, and 5 = strongly disagree). The mean score and percentage of each item were used for further analysis. The score for each item was calculated to obtain the mean score, with 0 corresponding to the least and 5 to the highest score (range: 0–5). For this purpose, the Likert scale was reverse coded to get a maximum score as strongly agreed (5 = strongly agree, 4 = agree, 3 = neutral, 2 = disagree, and 1 = strongly disagree). Furthermore, percentage agreement was the cumulative frequency of responses of those who “strongly agree” and “agree” with the barriers. Moreover, “strongly disagree”, “disagree”, and “neutral” were interpreted as negative responses. Section IV consisted of six statements evaluating the preparedness of community pharmacists towards the provision of public health services on a similar 5-point Likert scale (1 = strongly agree to 5 = strongly disagree). Furthermore, the *positive attitude* of pharmacists towards role enhancement in public health services was indicated by *percentage agreement*, with the cumulative frequency and percentages of those with “strongly agree” and “agree”, whereas, the *negative attitude* was depicted as “strongly disagree”, “disagree”, and “neutral”. For scoring purposes, reverse coding was performed to get the maximum score correlating with an excellent level of willingness. The score range for willingness was 1–5, i.e., 1 = unwillingness, 2 = poor willingness, 3 = average willingness, 4 = good willingness, and 5 = excellent level of willingness to participate in the future. Lastly, Section V had seven statements aimed to evaluate the opinion of community pharmacists on the effective strategies to enhance public health role on a similar 5-point Likert scale (1 = strongly agree to 5 = strongly disagree). Re-coding and scoring system in the present study was adapted from previously conducted research investigations [16,19].

### 2.5. Data Collection

The current study collected data by self-administration of the paper-based study tool. Data was scrutinized, converted to an Excel sheet, and subjected to further statistical analysis. All the responses were checked, and only completed responses were included. Incomplete responses were excluded from further analysis.

### 2.6. Statistical Analysis

Data analysis was performed using Statistical Package for the Social Sciences (SPSS) version 25. Descriptive data were summarized as frequency and percentages (%). Continuous data were expressed as Mean ± SD. The *p*-value of <0.05 was considered statistically significant. Chi-square/Fischer test was used to evaluate the association between the involvement of community pharmacists and demographics. Percentage agreement (%) to the statements assessing the participation of CPs in public health activities and barriers in the provision of these services, a 5-point Likert scale was converted to 2 points, i.e., “agree” and “strongly agree” was re-coded as “agreed” while “disagree”, “strongly disagree”, and “neutral” was converted to “disagreed”. Furthermore, to ascertain the significance and rank of the perceived barriers, relative importance index (RII) was used for ranking the barriers. RII equation was used, in which W = weight given to each statement by participant (5 = strongly agreed to 1 strongly disagreed), A = highest weight, and N = total number of participants. The highest value of RII (0 ≤ RII ≤ 1) corresponded to the main barrier.

$$RII=\frac{\sum W}{\left(A*N\right)}$$

## 3. Results

### 3.1. Characteristics of Study Participants

A total of 172 community pharmacists working in Al-Jouf province were approached for the survey. The response rate was more than half of the approached participants (n = 119/172; 69.2%). Of these 172 participants, 38 did not respond, and 15 questionnaires were incomplete and were excluded from the study. A total of 119 participants were included in the study. The mean age of the study participants was 32.2 ± 7.9 years. Most of the participants were males (n = 80/119, 67.2%), Saudi nationals (n = 79/119, 66.4%), having age 20–30 years (n = 65/119, 54.6%), had Bachelors in Pharmacy (B. Pharmacy) degree (n = 87/119, 73.1%), working as an employee (n = 96/119, 80.7%), in chain pharmacy setup (n = 87/119, 71.4%), resident pharmacists (n = 88/119, 73.9%), and had 1–5 years of work experience (n = 52/119, 43.7%). Detailed demographic features of the participants are shown in Table 1.

### 3.2. Involvement of Community Pharmacists in Public Health Services

The current study broadly categorized CPs’ participation into five major categories, i.e., lifestyle modification, screening services, awareness and counseling, drug-related information, and miscellaneous public health services. A total of 17 public health services were classified under the aforementioned categories, in Table 2, assessing the involvement of community pharmacists in public health services. The most practiced public health service in Al-Jouf was the provision of drug-related information, i.e., the provision of written information on drug use by pharmacists, whereas, the least practiced was the involvement in screening services.

The majority of the participants (n = 109/119, 91.6%) were aware of the public health services. Most of the participants were involved in at least one public health service (Table 2). Of 119 community pharmacists, majority were involved in providing drug use-related information (n = 114/119, 95.8%), counseling patients while dispensing medications (n = 108/119, 90.8%), referral of patients to government hospitals (n = 98/119, 82.4%), and creating awareness and counseling regarding vaccination/immunization (n = 96/119, 80.7%). Additionally, many CPs were involved in lifestyle modification and prevention of diseases such as screening for hypertension (n = 89/119, 74.8%) and weight management (n = 87/119, 73.1%). While the least (n = 87/119, 68.1%) were involved in screening for dyslipidemia, physical activity promotion (n = 81/119, 68.1%), and counseling of married couples while initiating treatment for sexually transmitted diseases (STDs) (n = 81/119, 68.1%).

Significant association of socio-demographic factors with public health services has been observed (Table 3). Identification of health-related risks in the community was practiced more by males (n = 56/91; 61.5%; *p*-value: 0.021) as compared to female pharmacists. The practice of certain public health services such as smoke cessation (n = 51/85; 60%; *p*-value: 0.027), healthy eating (n = 51/84; 60.7%; *p*-value: 0.026), screening for hypertension (n = 44/89; 49.4%; *p*-value: 0.024), and screening of infectious diseases (n = 41/82; 50%; *p*-value: 0.017) was more among young pharmacists between age 20–30 years. Saudi nationals were more involved in smoking cessation (n = 62/85; 72.9%; *p*-value = 0.020), immunization (n = 68/96; 70.8%; *p*-value: 0.049), counseling regarding contraception and ECP pills (n = 68/94; 72.3; *p*-value: 0.016), and the identification of health-related risks in the community (n = 67/91; 73.6%; *p*-value: 0.005). Participants with bachelor degrees tended to be more involved in smoke cessation (n = 64/85; 75.3%; *p*-value: 0.023) as compared to those pharmacists with higher degrees. Work experience of an average of 1–5 years (n = 39/82, 47.6%; *p*-value = 0.013) was found to be associated with screening of infectious diseases. Moreover, CPs working in chain pharmacies were significantly associated with screening for hypertension (n = 58/89, 65.2%; *p*-value = 0.010). Those who were employees in community pharmacies were more involved in screening of infectious diseases (n = 60/82; 73.2%; *p*-value: 0.002), counseling regarding STDs (n = 60/81; 74.1; *p*-value: 0.007), promoting AMSP (n = 59/82; 72%; *p*-value < 0.001), identification of health-related risks in the community (n = 69/91; 75.8%; *p*-value: 0.014), and referral to a government hospital (n = 75/98; 76.5%; *p*-value: 0.012) as compared to those pharmacists who owned pharmacies. The CPs filling less prescriptions (<50 per day) were significantly involved in smoke cessation (*p*-value: 0.038), physical activity promotion (0.005), and counseling while dispensing medication (n = 52/108; 48.1%; *p*-value: 0.023).

Overall, pharmacists of male gender, of 20–30 years of age, majorly Saudi nationals, having a bachelor of pharmacy degree and work experience of 1–5 years, working in chain pharmacies as employees, and filling <50 prescriptions per day were significantly involved in the provision of public health services.

On the contrary, the training level of CPs was not linked with any public health service. In other words, the public health services such as weight management, screening of diabetes, screening for dyslipidemia, providing written information on drug use, and personalized follow-up or private consultation were not statistically linked with any demographic characteristic of CPs.

### 3.3. Barriers in Providing Public Health Services

Fourteen items were included in the present study that were potential barriers for pharmacists in delivering public health services (Table 4). All of the items in barriers reported mean scores greater than three. The highest ranked barrier reported that hindered CP’s involvement in public health services was “*patients do not give time for such services*” (RI = 0.812; Mean score = 4.06 ± 0.85; 79%) followed by “*lack of coordination with other healthcare professionals*” (RI = 0.802; mean score = 4.01 ± 0.85; 76.5%)*,* “*lack of access to training programs on public health*” (RI = 0.802; mean score = 4.01 ± 0.89; 75.6%), and “*shortage of pharmacy assistants or technicians*” (RI = 0.797; mean score = 3.98 ± 0.98; 78.2%). However, the lowest ranked barrier reported by CPs was a “*lack of confidence in my ability/level of information acquired by practicing pharmacists*” (RI: 0.748; mean score = 3.74 ± 1.15; 68.1%). The second lowest ranked barrier was “*patients generally have more urgent medical conditions*” (RI: 0.751; mean score = 3.76 ± 0.88; 68.9%). The third lowest ranked barriers were “*lack of financial compensation*” (RI: 0.761; mean score = 3.81 ± 1.00; 68.9%) and “*lack of patient demand for these services*” (RI: 0.761; mean score = 3.81 ± 0.91; 64.7%). The male gender tends to agree more with the barriers to involvement as compared to the female gender (Table 4); however, this gender association is not statistically significant. The association between the level of training of CPs with the perceived barriers was statistically significant. Resident pharmacists tend to agree that lack of clinical tools (n = 69/91; 75.8%; *p*-value: 0.041) and lack of coordination with other healthcare professionals (n = 667/91; 73.6%; *p*-value: 0.041) were the main barriers to the CPs involvement in public health services.

### 3.4. Preparedness of Community Pharmacists towards Public Health Services

The willingness of pharmacists to incorporate themselves into public health services was assessed using six statements. All of the items corresponding to the preparedness of CPs reported mean scores greater than four for the majority of the statements, indicating a good level of preparedness of CPs (Table 5). A higher proportion (79%) of participants reported a *positive attitude* by agreeing that *pharmacists in general are willing to provide public health services*, supplementing with the mean score of 4.19 ± 0.78, indicating a satisfactory level of preparedness. Association of gender with the preparedness of CPs’ involvement was more in the male gender as compared to the females (Table 5); however, this gender association is not statistically significant. There was an association between the level of preparedness and level of training of CPs (*p*-value: 0.037). Resident pharmacist indicated higher levels of preparedness (n = 57/79; 72.2%) where majority of them agreed that their pharmacies are prepared to provide public health services.

### 3.5. Strategies to Enhance the Public Health Role of Community Pharmacists

There were seven proposed strategies to enhance the role of CPs in public health services (Table 6). The majority of the pharmacists agreed that “*empowerment through education and awareness*” (n = 100/119; 84%), *teaching the use of new technologies* (n = 99; 83.2%), and *teaching macro-level public health activities* (n = 99/119; 83.2%) will be the most effective techniques in improving the role of CPs in public health. The association of gender with strategies to improve CPs role was significant for the *teaching of macro-level public health activities* strategy, with the male gender agreeing more than the female gender (n = 71/99; 71.7%; *p*-value: 0.034). However, there is no significant association between the training level of CPs and their agreement to strategies for the enhancement of the future role of pharmacists.

## 4. Discussion

The current study is the first of its kind, assessing the outreach of community pharmacists in public health services in Al-Jouf, a remote region in Saudi Arabia with limited access to healthcare facilities. The findings of the present study documented that the majority of the registered pharmacists working in such remote vicinity had a good level of understanding of public health services. Overall, these findings reflect the shift from traditional compounding and dispensing services towards extended roles of community pharmacists in public health. These findings were in line with the studies conducted across the globe [2,20,21]. Previously conducted studies in Saudi Arabia also coincide with these findings [22]. However, there is a dearth of actual data from other remote regions, as the majority of the studies were from big cities. Additionally, this study evaluated the level and extent of preparedness of pharmacists to extend their roles in achieving the Saudi Vision 2030, not only in metropolitan cities but all over the country.

Interestingly, most of the pharmacists were aware of public health services in the current study. This adequate awareness of pharmacists regarding public health services at the community level corroborates with the findings of studies conducted in Pakistan, Scotland, and Nigeria reporting 99%, 90%, and 80% awareness, respectively [16,18,20,23]. Outcomes of the present study were more inclined toward CPs’ role in the provision of drug use-related information, patient counseling while drug-dispensing, and creating awareness regarding vaccination. These are similar to other previously conducted studies in Malaysia and Pakistan [16,24]. On the contrary, it is pertinent to note that in developed nations, CPs were more likely involved in screening and lifestyle modification services, including screening of chronic diseases such as diabetes and hypertension [25,26,27,28,29,30]. Additionally, screening for dyslipidemia was considerably low in the present study but still did not corroborate with the studies conducted in Pakistan and Canada, in which extremely low (i.e., 4.8% and 6.5%, respectively) number of CPs were practicing dyslipidemia screening [16,31].

Despite the awareness of pharmacists in public health services, the majority were not involved in the provision of certain services, particularly in lifestyle modifications to prevent diseases and improve patient health outcomes. This lack of involvement is an indication that factors are hindering the provision of these services. To overcome these barriers, various strategies will need to optimize the outcome and further enhance CPs’ role in public health services. The most eminent and highest-ranked barrier reported in this study indicated that patients have insufficient time to avail these services offered by the CPs, aligning with the results of the previous study [16]. Moreover, these findings highlight the fact that the highest ranked barrier is not due to the inefficiency of CPs, but due to the lack of time patients have to avail the expert opinion on drugs and health related issues of the pharmacists. This could also be due to unawareness of the general public about the significance and potential benefits of pharmacist-led counseling to achieve health goals. To eradicate this barrier, awareness campaigns and promotional techniques regarding counseling by pharmacists would immensely help the end-user. Our study also documented similar strategies to be the most effective, i.e., awareness and educational campaigns similar to those recommended in studies conducted in Australia, USA, and Pakistan [16,32,33].

The current study identified that there is insufficient communication with other healthcare professionals as the 2nd highest-ranked barrier. These findings are in agreement with the results of previously conducted studies in Indonesia, Canada, and Pakistan [16,34,35]. Promoting collaboration of pharmacists with other healthcare professionals via the introduction of mandatory training sessions and ward rounds (if working at hospital levels), via telecommunication (providing information about the nearby hospitals and clinics) to bridge the gap will help in eradicating this miscommunication. This strategy will eventually lead to improved patient care by integration of CPs in the system [16,33]. Another significant barrier was highlighted in the current study was lack of appropriate training programs that need to be organized to update the knowledge and skills of the pharmacists working in community setups. The similar results have been reported by other studies [16,34,35]. On-job training sessions, conferences, and online training sessions must be organized by pharmacist working in public and private sectors, keeping in mind the continuous recurrence of COVID pandemic waves. However, the present study reported a statistically significant association of resident pharmacists with the reporting of this barrier. This might indicate that at the residency level, resident pharmacists have limited or no access to healthcare professionals. For this reason, CPs must be trained and provided access to form liaisons with other healthcare workers. A interdisciplinary training program will also help to eradicate this barrier.

Less number of pharmacy assistants or pharmacy technicians was identified as third most reported barrier. This indicated that due to increased workload, CPs are unable to participate in public health services, and their role is limited to traditional services. These findings are in line with a survey conducted in Nigeria [36]. Contrarily, it does not coincide with a study conducted in Pakistan that does not recognize the shortage of technical staff [16]. These results necessitate a dire need to have pharmacy technicians according to the workload of the pharmacies. In this context, the health authorities can design framework to determine the number of pharmacy technicians required by a pharmacy in order to facilitate the pharmacists for public health services. This barrier is frequently reported by male and resident pharmacists in this study. However, this could be due to the higher number of male participants in our study.

Regarding the preparedness of CPs to get involved in provision of public health services, this study indicated a satisfactory level of willingness and positive attitude of CPs toward their involvement in public health services. These results corroborate with previous studies conducted in China, USA, Pakistan, and Nigeria [16,37,38,39] where CPs showed positive attitudes and preparedness for public health services. These findings reflected that CPs have adequate clinical experience which could be no obstacle in the provision of public health services, resonating with their competency. However, the resident pharmacists were significantly more inclined, and they were currently prepared to provide public health services. On the other hand, there was no statistically significant association between gender and level of preparedness for public health services.

Current study also evaluated plausible strategies that would enhance the involvement of CPs in public health services. Empowerment through education and awareness was the most recommended strategical technique recommended by the CPs. These recommendations are similar to other studies conducted in various parts of the world. Other popularly documented strategies by CPs included incorporating teaching and training sessions demonstrating the use of new technologies at macro-level public health activities, hence, extending and enhancing the role of community pharmacists. Interestingly, the female gender tends to be significantly associated with the encouragement of the introduction of new technologies at the macro level.

It is important to note that Saudi pharmacists were almost double in proportion as compared to non-Saudi pharmacists that might be a possible reason for efficient delivery of the public health services in this study. Previous studies have indicated that expats being unaccustomed with the culture and having language barrier are less involved in public health services. Contrary to the previous studies, current findings highlighted that a major proportion of the CPs were employees rather than self-employed as compared to a previous survey in KSA where the majority were owners and hence more business-oriented than patient-centered [40]. However, it was consistent with the findings of a study conducted in Pakistan where 84.8% of pharmacists were working as employees [16]. These two factors could be linked to the difference in public health services provision to such an extent in a remote area such as Al-Jouf. Furthermore, it is pertinent to note that various other factors may limit and facilitate the provision of public health services in community pharmacies that are not ascertained by the current study such as lack of updated knowledge and skills, lack of recognition, trust on CP’s knowledge and skills at the patient level, and inaccessibility to the medical records of patients.

### 4.1. Recommendations for Stakeholders

The fact is acknowledged that the Ministry of Health of Saudi Arabia is in the continuous process of updating the regulations regarding the provision of public health services at the community level [30]. However, to achieve the Saudi Vision 2030, implementation needs to be expedited. Pharmacists working at the community level must be well equipped in every aspect such as updated medical, pharmaceutical, and clinical knowledge, competitive skills, proper professional qualification, and good managerial skills to subdue perceived barriers with a proper sense of responsibility. However, currently, the Saudi health authorities have a paucity of working guidelines in place for community pharmacists to diligently carry out their recommended duties [41]. Hence, there is a dire need at the policy making and implementation level. The policymakers such as the Ministry of Health, Saudi Food and Drug Authority (SFDA), Saudi Pharmaceutical Society (SPS), Saudi Patient Safety Center (SPSC), and other Pharmacists associations in the country should collaborate and achieve the standard operating procedures (SOPs) being practiced worldwide [42,43,44]. Continued medical education, periodic training sessions, and refresher on-job courses must be designed, conducted, and made mandatory for practicing by relevant professional organizations to help the CPs remain up-to-date and efficient in performing their roles [30,45,46]. If implemented at national level, CPs services will be standardized and eventually will help to achieve the Saudi Vision 2030 efficiently. These findings also encourage global pharmacist organizations to focus on extended roles of CPs. Pharmacists led public health services should be integrated into pharmacy curriculum and residency programs. There is a need of collaboration between public health and pharmacy organizations at global level.

### 4.2. Limitations and Strengths of the Study

The present study is limited by a smaller sample size due to the limited pharmacies in Al-Jouf, which could be a potential bias, followed by the COVID pandemic still being present and possibly fewer pharmacists working at the time of data collection. Additionally, there is a strong probability that the pharmacists who participated in the study could be different and respond differently from those who did not participate, i.e., as only pharmacists in Al-Jouf pharmacies were approached. Moreover, a convenient sampling technique was used for data collection, which limits the generalizability of the findings. Furthermore, even though the association between the variables might exist, due to the cross-sectional study design, we were unable to establish causal inferences. This study was conducted in a single region and might not be extrapolated to other regions having varying CPs’ practicing cultures. Additionally, the recruited participants may have the bias of being part of the population who was less inclined to participate in such activities. Nevertheless, this study is strengthened by being the first representation of the estimation of CPs’ participation in public health services in the Al-Jouf region in Saudi Arabia. Furthermore, previous studies did not compile and discuss data of both Saudi nationals and non-Saudi nationals, which is another strength of this survey.

### 4.3. Implications of the Current Study for Practice

The motivators to improve the participation of CPs in public health services might include incentives and rewards, which could be vital for the shift towards patient-oriented practice at the community pharmacy level [47]. Furthermore, there is a need to conduct qualitative analysis in the future to better evaluate the specific factors influencing the participation of community pharmacists towards public health services, both barriers and facilitators. Specifically, the current study will improve health outcomes by enhancing CPs’ role in remote regions with meager health services and restricted access to them, such as Al-Jouf. Making strategies to overcome these barriers is the utmost need of the hour and the responsibility of concerned health authorities to substantiate the role of pharmacists working in community pharmacies regarding participation in public health services. Furthermore, the findings of this study can be contemplated for various similar regions like Al-Jouf. These outcomes will also help in increasing general clinical pharmacy services and specialized ambulatory care services [11].

## 5. Conclusions

The present study underscored the adequate involvement of pharmacists in public health services in remote regions of the KSA. Study recruits reflected a higher and satisfactory inclination towards future participation in current and expanding practices. Additionally, continued education will prove to be beneficial in further involvement of pharmacists in the activities in which they were less likely to be involved currently. Furthermore, pharmacists should self-participate and play a proactive role for the betterment of their profession and enhancement of their role. Moreover, further studies will be required to evaluate the outcomes of the recommended strategies provided in the current study to further enhance the role of community pharmacists. Finally, policies should be in place and strategies to implement these policies and integrate pharmacists in more clinical and public health roles should be expedited by the relevant health authorities.

## Figures and Tables

**Table 1:** Demographic characteristics of the study participants (N = 119).

Variables	Frequency (%)
**Gender**	
Male	80 (67.2)
Female	39 (32.8)
**Age**	
20–30 years	65 (54.6)
31–40 years	40 (33.6)
>40 years	14 (11.8)
**Nationality**	
Saudi	79 (66.4)
Non-Saudi	40 (33.6)
**Qualification**	
Bachelors in Pharmacy	87 (73.1)
Doctor of Pharmacy	23 (19.3)
MPhil/MS	6 (5)
Ph.D.	3 (2.5)
**Level of training**	
Resident (Pharmacist)	88 (73.9)
Associated Consultant (Senior Pharmacist)	13 (10.9)
Consultant	18 (15.1)
**Work experience**	
≤5 years	59 (49.6)
6–10 years	37 (31.1)
11–15 years	13 (10.9)
>15 years	10 (8.4)
**Type of community pharmacy**	
Chain Pharmacy	85 (71.4)
Independent pharmacy	34 (28.6)
**Average prescriptions filled per day**	
<50	62 (52.1)
51–100	30 (25.2)
>100	27 (22.7)
**Pharmacy ownership**	
Employee	96 (80.7)
Owner	23 (19.3)

**Table 2:** The extent of involvement of community pharmacists in public health services (N = 119).

Public Health Services	Yes n (%) *	No n (%)
**Lifestyle modification**		
Weight management	87 (73.1)	32 (26.9)
Smoking cessation	85 (71.4)	34 (28.6)
Physical activity promotion	81 (68.1)	38 (31.9)
Healthy eating	84 (70.6)	35 (29.4)
**Screening services**		
Screening for diabetes	83 (69.7)	36 (30.3)
Screening for hypertension	89 (74.8)	30 (25.2)
Screening for dyslipidemia	81 (68.1)	38 (31.9)
Screening of infectious diseases	82 (68.9)	37 (31.1)
**Awareness and counseling**		
Vaccination and immunization	96 (80.7)	23 (19.3)
Counseling regarding treatment for sexually transmitted diseases (STDs)	81 (68.1)	38 (31.9)
Counseling on emergency and other contraception pills	94 (79)	25 (21)
**Drug-related information**		
Counseling while dispensing medications	108 (90.8)	11 (9.2)
Providing written information on drug use	114 (95.8)	5 (4.2)
Personalized follow-up or private consultation	92 (77.3)	27 (22.7)
Promote antimicrobial stewardship programs	82 (68.9)	37 (31.1)
**Miscellaneous Public Health Services**		
Assessment to identify the health-related risks in community	91 (76.5)	28 (23.5)
Referral to government hospital	98 (82.4)	21 (17.6)

* n = frequency.

**Table 3:** Association of public health services with the demographics among community pharmacists.

Public Health Service	Gender	Age	Nationality	Qualification	Level of Training	Work Experience	Type of Community Pharmacy	Ownership of Pharmacy	Prescriptions Filled per Day
Weight management	0.379	0.113	0.081	0.150	0.120	0.535	0.069	0.610	0.200
Smoking cessation	0.086	**0.027**	**0.020**	**0.023**	0.387	0.062	0.654	0.802	**0.038**
Physical activity promotion	0.676	0.095	0.097	0.811	0.613	0.243	0.892	0.217	**0.005**
Healthy eating	0.085	**0.026**	0.203	0.231	0.178	0.068	0.081	1.000	0.252
Screening for diabetes	0.673	0.223	0.291	0.340	0.154	0.745	0.381	0.206	0.292
Screening for hypertension	1.000	**0.024**	0.117	0.169	0.629	0.959	**0.010**	0.059	0.917
Screening for dyslipidaemia	0.302	0.053	0.407	0.056	0.670	0.954	1.000	0.322	0.234
Screening of infectious diseases	0.095	**0.017**	0.063	0.573	0.137	**0.013**	0.051	**0.002**	0.750
Vaccination and immunisation	0.322	0.531	**0.049**	0.744	0.261	0.183	0.212	0.239	0.171
Counseling regarding STDs treatment	0.676	0.514	0.097	0.293	0.330	0.210	0.278	**0.007**	0.953
Counseling on ECP and other contraception pills	0.154	0.259	**0.016**	0.266	0.738	0.121	0.330	0.154	0.796
Counseling while dispensing medications	0.337	0.245	0.504	0.164	0.944	0.211	0.727	0.220	**0.023**
Providing written information on drug use	1.000	0.753	0.333	0.934	0.188	0.651	0.320	1.000	0.640
Personalized follow-up or private consultation	0.102	0.717	0.246	0.592	0.849	0.346	0.231	0.097	0.565
Promote AMSP	0.212	0.052	**0.023**	0.679	0.797	0.486	0.051	**<0.001**	0.641
Identification of the health-related risks in community	**0.021**	0.367	**0.005**	0.444	0.717	0.081	0231	**0.014**	0.332
Referral to government hospital	0.201	0.222	0.202	0.567	0.162	0.422	1.000	**0.012**	0.526

The table shows the *p*-value obtained from the chi-square test; *p*-value of less than 0.05 is considered statistically significant and is represented in bold, STD: Sexually transmitted diseases, ECP: Emergency contraceptive pill, AMSP: Antimicrobial stewardship programs.

**Table 4:** Association of barriers in providing public health services with gender and level of training of community pharmacists (N = 119).

Barriers	Mean Score ^a^	% Agreement ^b^ n (%)								RII ^c^	Ranking ^d^
		Total	Gender			Level of training of CP					
			Male	Female	*p*-Value *	RP	SP	C	*p*-Value **		
Lack of knowledge or clinical skills	3.87 ± 1.06	86 (72.3)	61 (70.9)	25 (29.1)	0.193	64 (74.4)	11 (12.8)	11 (12.8)	0.347	0.775	6
Shortage of time	3.93 ± 0.81	91 (76.5)	60 (65.9)	31 (34.1)	0.651	65 (71.4)	12 (13.2)	14 (15.4)	0.339	0.787	4
Shortage of pharmacists	3.88 ± 1.02	84 (70.6)	53 (63.1)	31 (36.9)	0.198	63 (75)	11 (13.1)	10 (11.9)	0.199	0.776	5
Shortage of pharmacy assistants or technicians	3.98 ± 0.98	93 (78.2)	62 (66.7)	31 (33.3)	1.000	70 (75.3)	11 (11.8)	12 (12.9)	0.405	0.797	3
Lack of confidence in my ability/level of information	3.74 ± 1.15	81 (68.1)	57 (70.4)	24 (29.6)	0.302	63 (77.8)	6 (7.4)	12 (14.8)	0.184	0.748	10
Patients do not give time for such services	4.06 ± 0.85	94 (79)	62 (66)	32 (34)	0.638	70 (74.5)	11 (11.7)	13 (13.8)	0.683	0.812	1
Lack of patient demand for these services	3.81 ± 0.91	77 (64.7)	54 (70.1)	23 (29.9)	0.416	55 (71.4)	11 (14.3)	11 (14.3)	0.280	0.761	8
Lack of financial compensation	3.81 ± 1.00	82 (68.9)	57 (69.5)	25 (30.5)	0.527	58 (70.7)	12 (14.6)	12 (14.6)	0.155	0.761	8
Lack of space in the pharmacy for such services	3.87 ± 0.95	84 (70.6)	57 (67.9)	27 (32.1)	0.833	63 (75)	11 (13.1)	10 (11.9)	0.199	0.773	7
Patients generally have more urgent medical conditions	3.76 ± 0.88	82 (68.9)	55 (67.1)	27 (32.9)	1.000	61 (74.4)	11 (13.4)	10 (12.2)	0.223	0.751	9
Legal and regulatory constraints/unclear regulatory environment	3.87 ± 0.85	82 (68.9)	59 (72)	23 (28)	0.139	60 (73.2)	11 (13.4)	11 (13.4)	0.363	0.773	7
Lack of clinical tools	3.93 ± 0.89	91 (76.5)	63 (69.2)	28 (30.8)	0.491	69 (75.8)	12 (13.2)	10 (11)	0.041	0.787	4
Lack of coordination with other healthcare professionals	4.01 ± 0.85	91 (76.5)	63 (69.2)	28 (30.8)	0.491	67 (73.6)	13 (14.3)	11 (12.1)	0.041	0.802	2
Lack of access to training programs on public health	4.01 ± 0.89	90 (75.6)	61 (67.8)	29 (32.2)	0.823	69 (76.7)	11 (12.2)	10 (11.1)	0.087	0.802	2

CP: community pharmacist, RP: resident pharmacist, SP: senior pharmacist, C: consultant pharmacist, ^a^ Mean score ± SD on Likert scale: strongly disagree = 1; disagree = 2; neutral= 3; agree = 4; strongly agree = 5; SD: standard deviation; ^b^ Percentage agreement response is the summation of agree/strongly agree options, ^c^ RI: relative index, ^d^ Ranking according to the RII ((0 ≤ RII ≤ 1)), * *p*-value obtained from chi-square test showing an association between barriers and genders; *p*-value < 0.05 is significant, ** *p*-value obtained from chi-square test showing an association between barriers and level of training of community pharmacists.

**Table 5:** Preparedness of community pharmacists towards public health services and their association with gender and level of training of community pharmacists (N = 119).

Statement	Mean Score ^a^ (Mean ± SD)	Percentage Agreement ^b^ n (%)							*p*-Value **
		Total	Gender		*p*-Value *	Level of Training of CP			
			Male	Female		RP	SP	C	
I feel that I have enough clinical knowledge to provide public health services	4.07 ± 0.89	87 (73.1)	61 (70.1)	26 (29.9)	0.279	63 (72.4)	8 (9.2)	16 (18.4)	0.195
I feel that I have enough clinical experience to provide public health services	4.03 ± 0.78	91 (76.5)	64 (70.3)	27 (29.7)	0.250	66 (72.5)	9 (9.9)	16 (17.6)	0.363
My pharmacy is currently prepared to provide public health services	3.87 ± 0.93	79 (66.4)	55 (69.6)	24 (30.4)	0.536	57 (72.2)	6 (7.6)	16 (20.3)	0.037
I think pharmacists in general are willing to provide public health services	4.19 ± 0.78	94 (79)	64 (68.1)	30 (31.9)	0.811	68 (72.3)	10 (10.6)	16 (17)	0.535
I have sufficient references to provide public health services in daily practice	4.02 ± 0.94	90 (75.6)	60 (66.7)	30 (33.3)	1.000	65 (72.2)	9 (10)	16 (17.8)	0.340
I am satisfied with my role as community pharmacist	3.95 ± 1.01	86 (72.3)	57 (66.3)	29 (33.7)	0.829	59 (68.6)	12 (14)	15 (17.4)	0.086

CP: community pharmacist, RP: resident pharmacist, SP: senior pharmacist, C: consultant pharmacist, SD: standard deviation; ^a^ Mean score ± SD on Likert scale: strongly disagree = 1; disagree = 2; neutral = 3; agree = 4; strongly agree = 5; ^b^ Percentage agreement response is the summation of agree/strongly agree options; * *p*-value obtained from chi-square test showing an association between strategies and gender; *p*-value < 0.05 is significant; ** *p*-value obtained from chi-square test showing an association between strategies and level of training of community pharmacists.

**Table 6:** Strategies to enhance community pharmacists` involvement in public health services and their association with gender and level of training of community pharmacists.

Strategies to Improve CPs’ Involvement in Public Health Services in Future	Percentage Agreement ^a^ n (%)							
	Total	Gender		*p*-Value *	Level of Training of CPs			*p*-Value **
		Male	Female		RP	SP	C	
Empowerment through education and awareness	100 (84)	67 (67)	33 (33)	1.000	72 (72)	10 (10)	18 (18)	0.121
Empowerment through direct remuneration of pharmacists	95 (79.8)	63 (66.3)	32 (33.7)	0.809	68 (71.6)	11 (11.6)	16 (16.8)	0.482
The use of new technologies and social media in practice	96 (80.7)	63 (65.6)	33 (34.4)	0.622	69 (71.9)	10 (10.4)	17 (17.7)	0.273
Teaching the use of new technologies	99 (83.2)	67 (67.7)	32 (32.3)	0.800	73 (73.7)	10 (10.1)	16 (16.2)	0.675
Encouraging collaboration between pharmacists and other healthcare professionals	97 (81.5)	68 (70.1)	29 (29.9)	0.209	72 (74.2)	9 (9.3)	16 (16.5)	0.376
Tackling a number of barriers	96 (80.7)	66 (68.8)	30 (31.3)	0.469	71 (74)	10 (10.4)	15 (15.6)	0.905
Teaching macro-level public health activities	99 (83.2)	71 (71.7)	28 (28.3)	0.034	74 (74.7)	10 (10.1)	15 (15.2)	0.812

CP: community pharmacist, RP: resident pharmacist, SP: senior pharmacist, C: consultant pharmacist, SD: standard deviation; ^a^ Responses of the participants who agreed and strongly agreed are included; * *p*-value obtained from chi-square test showing an association between gender with CPs’ preparedness towards involvement in public health services; *p*-value < 0.05 is considered significant; ** *p*-value obtained from chi-square test showing an association between level of training and preparedness of CPs towards involvement in public health services.

## Data Availability

All underlying data are presented in this manuscript.
